# Surface Activation Using Atmospheric Plasma to Improve PHB Coating Adhesion and Corrosion Resistance of AZ91D Magnesium Alloys

**DOI:** 10.3390/polym18020205

**Published:** 2026-01-12

**Authors:** Arturo Valenzo, María del Pilar Rodríguez-Rojas, Horacio Martínez, Victoria Bustos-Terrones, Alvaro Torres-Islas, Socorro Valdez, Arturo Molina-Ocampo

**Affiliations:** 1 Facultad de Ciencias Químicas e Ingeniera (FCQeI), Universidad Autonóma del Estado de Morelos, Cuernavaca 62210, Morelos, Mexico; alvaro.torres@uaem.mx; 2 Spectroscopy Laboratory, Institute of Physical Sciences, National Autonomous University of Mexico, Cuernavaca 62210, Morelos, Mexico; mpilar@icf.unam.mx (M.d.P.R.-R.); svaldez@icf.unam.mx (S.V.); 3 Environmental Engineering and Sustainability Research Laboratory, Polytechnic University of the State of Morelos, Boulevard Cuauhnáhuac 566, Col. Lomas del Texcal, Jiutepec 62574, Morelos, Mexico; vbustos@upemor.edu.mx; 4 Centro de Investigaciones en Ingeniería Aplicadas (CIICAp-UAEM), Av. Universidad 1001, Cuernavaca 62209, Morelos, Mexico; arturo_molina@uaem.mx

**Keywords:** Mg, AZ91D, PHB polymer, plasma processing

## Abstract

Polyhydroxybutyrate (PHB) is considered a coating material capable of limiting the corrosion of biodegradable metallic implants due to its biocompatibility and ability to form a physical barrier. In this study, PHB was deposited on commercial AZ91D magnesium alloy using the spin coating technique. To improve adhesion at the polymer–substrate interface, the magnesium substrates were subjected to atmospheric pressure plasma treatment for different exposure times (5, 10, or 15 min) before coating. The optimal treatment time of 5 min significantly increased substrate wettability and surface free energy, facilitating stronger PHB adhesion. In addition, the PHB coatings were subjected to atmospheric pressure plasma treatment for 5, 10, or 15 s to evaluate potential surface modifications. Corrosion behavior under simulated physiological conditions was assessed via potentiodynamic polarization and electrochemical impedance spectroscopy (EIS) in HANK’s solution at 37 °C. Pull-off tests were used to evaluate the adhesion strength between the coating and the substrate under each treatment condition. The results showed a significant decrease in the corrosion rate (V_corr_), from 4.083 mm/year for bare Mg-AZ91D to 0.001 mm/year when both the substrate and the polymer received plasma treatment. This indicates that the treatment modifies surfaces and improves interfacial bonding, enhancing polymer–metal interaction and producing durable, biocompatible coatings for medical implants.

## 1. Introduction

Magnesium is considered a promising biomaterial due to its biocompatibility, mechanical properties, and biodegradability. This metal’s elastic modulus of 45 GPa is close to that of human bone (3–20 GPa), reducing the risk of damage from mechanical incompatibility. Furthermore, it is non-toxic to humans, with a recommended daily intake considerably higher than that for other metals used in implants (240–420 mg/day) [1]. The study of magnesium alloys in biomaterials research has gained relevance due to their properties being comparable to those of natural bone. However, their application in orthopedic implants is limited by their high corrosion rate, which can lead to a rapid loss of mechanical integrity and compromise long-term clinical performance [2]. Thus, it is necessary to develop strategies that improve the corrosion resistance of magnesium-based implants to enable their safe clinical use. Among the available approaches, surface modification with protective coatings is recognized as an effective method to reduce corrosion [3].

In this context, polyhydroxyalkanoates (PHAs) represent a family of microbial polyesters with thermoplastic behavior. They are biodegradable, biocompatible, and suitable for a wide range of biomedical applications [4]. Among them, poly(3-hydroxybutyrate) (PHB) stands out for its high crystallinity (over 60%), biocompatibility, and good interaction with biological tissues, making it a promising material for coating magnesium alloys. Additionally, it has a low glass transition temperature (below 5 °C) and produces minimally acidic degradation products. These characteristics position PHB as a promising and durable polymer coating for application on magnesium alloys [5].

The surface properties of modern materials are often inadequate for direct applications, exhibiting deficiencies in wettability, adhesion, and biocompatibility. Therefore, it is necessary to modify them beforehand by adjusting both morphology and chemical composition to achieve the desired functional surface [6]. Several techniques have been explored for surface modification, and atmospheric pressure plasma treatment has gained relevance in biomedical applications due to its operational simplicity and relatively low environmental impact. Non-equilibrium plasma is generated when the temperature of electrons is moderately high in comparison to that of ions, atoms, and molecules. The main effect of this plasma is the generation of reactive oxygen and nitrogen species, which can modify the surface chemistry and microstructure of materials. These reactive species induce significant changes in the surface chemistry and microstructure of the polymer, promoting the formation of polar functional groups and increasing surface energy. In this context, this paper emphases studying the performance, for the first time, a dual-time-scale atmospheric plasma strategy that independently optimizes magnesium alloy and PHB surfaces, establishing a direct correlation between plasma-induced surface chemistry, polymer crystallinity, interfacial adhesion, and corrosion protection, resulting in a simple yet highly efficient biodegradable coating system [7].

## 2. Materials and Methods

### 2.1. Preparation of AZ91D Magnesium Alloy (Mg-AZ91D)

Mg-AZ91D was purchased from Ningbo Picado Trading Co., Ltd., Ningbo, China, in the form of 15.0 × 15.0 × 0.5 cm^3^ plates, from which twelve specimens measuring 3.0 × 3.0 × 0.5 cm^3^ were cut. Abrasive papers with grit numbers ranging from 120 to 600 were used to obtain homogeneous surfaces. The specimens were cleaned with ethanol in an ultrasonic bath (SC Scientific, Warminster, PA, USA) for 20 min. Afterwards, they were dried in an oven (Rorer, Mexico City, Mexico) at 40 °C.

### 2.2. Preparation of Polyhydroxybutyrate (PHB)

The polymer used was obtained from the Institute of Biotechnology at UNAM, Morelos, México in the form of flakes with a molecular weight of 1441 kDa. The flakes were ground using a mortar. Four grams of PHB were weighed and 100 mL of chloroform was added. The polymer solution was placed on a hot plate at 40 °C with stirring at 200 rpm for 2 h.

### 2.3. Preparation of Coatings

Three milliliters of PHB solution were measured and deposited onto magnesium substrates using a spin coating system operating at 300 rpm for 3 min. This procedure was performed following the methodology previously reported in a published article to ensure reproducibility and direct comparison of the results. Under these conditions, the average thickness of the resulting film was 15 ± 1 μm [8].

### 2.4. Atmospheric Plasma Treatment

The Atmospheric plasma treatment was done using an APC 500 plasma source (Diener Electronics, Ebhausen Germany). The experimental conditions were voltage of 20 kV, 25 mA of discharge current, at 40 kHz of frequency, and air pressure of 1500 Torr. The plasma discharge was created by two electrodes. The discharge expanded from the electrode zone due to air flows and the active plasma area was ~10 cm^2^. We previously reported the characterization of the plasma discharge using optical emission spectroscopy (OES) [9]. The species observed from lowest to highest intensity were O●, N_2_+, N_2_, and ●OH. An electron temperature of (0.36 ± 0.04) eV and electron density of (1.95 ± 0.24) × 10^12^ cm^−3^. The treated samples had dimensions of 3.0 × 3.0 × 0.5 cm^3^ and were placed 4 cm from the plasma outlet within the most homogeneous zone at the center of the plasma region. Atmospheric plasma treatment under static conditions was applied to the surface of bare Mg-AZ91D for 5, 10, or 15 min and, separately, to PHB-coated Mg-AZ91D samples for 5, 10, or 15 s. In the latter case, the polymer was already deposited on the Mg substrate, allowing the evaluation of plasma effects on the coated system independently from the bare substrate. This approach enabled the assessment of to what extent the plasma treatment on both the metallic substrate and the polymer-coated samples improved surface properties such as adhesion, wettability, and corrosion resistance.

### 2.5. Sessile Drop Technique

The wettability of Mg-AZ91D and PHB was measured using the sessile drop technique by recording the contact angle to calculate the surface free energy. Five microliters of distilled water and five microliters of diiodomethane were used. Six measures in different zones of the sample surface were performed, and the standard error was obtained from the standard deviation statistical analysis. To calculate the surface free energy and its polar and dispersive components, the Owens–Wendt mathematical model was applied following the method described in the literature. This model is used to determine the surface energy of solids by analyzing the contact angle of a liquid droplet on the material surface [10]. In this study, distilled water and diiodomethane (99% purity), both supplied by Sigma-Aldrich (St. Louis, MO, USA), were used.

### 2.6. Pull-Off Tests

Adhesion tests were performed according to ASTM D4541. The samples consisted of magnesium coated with PHB subjected to different plasma treatment durations. The tests were conducted using a Defelsko pull-off adhesion tester (DeFelsko Corporation, Ogdensburg, NY, USA).

### 2.7. X-Ray Diffraction (XRD)

X-ray diffraction analysis was carried out to identify the crystalline phases present on the surface of the Mg-AZ91D alloy. Measurements were performed using a Rigaku Miniflex DMAX 2200 XRD diffractometer (Rigaku Corporation, Japan) equipped with Cu-Kα radiation (λ = 1.5406 Å). The scan range was 2θ = 10° to 90°. For the PHB coatings, we used Cu-Kα radiation (λ = 1.5406 Å) in grazing incidence mode over a 2θ range of 10° to 30°, aiming to analyze the polymer’s crystalline structure. The crystallite size (Xs) of PHB was calculated using the Scherrer equation.

Xs = Kλ/βcosθ(1)

### 2.8. Raman Spectroscopy

Raman spectroscopy was employed to identify the characteristic functional groups of the PHB coating applied to magnesium alloy. Analyses were performed using a SENTERRA II spectrometer (Bruker Corporation, Billerica, MA, USA) equipped with a 785 nm excitation laser. Spectra were acquired in the range of 400 to 3000 cm^−1^, and each measurement was conducted with an integration time of 30,000 ms.

### 2.9. Potentiodynamic Polarization Curves and Electrochemical Impedance Spectroscopy

For the electrochemical characterization, a three-cell electrode configuration was used with a Gill AC potentiostat (ACM Instruments, Cumbria, UK). An Ag/AgCl electrode served as the reference, a platinum mesh was employed as the counter electrode, and the sample acted as the working electrode. The geometric area of the sample exposed to the electrolytic medium was 1 cm^2^. Two sets of experiments were conducted to evaluate the effect of the atmospheric pressure plasma treatment on corrosion behavior. In the first set, uncoated Mg-AZ91D samples were evaluated, including the untreated alloy and specimens treated with plasma for 5, 10, or 15 min. Electrochemical impedance spectroscopy (EIS) was measured in the frequency range of 10 KHz to 0.01 with a 15 mV sinusoidal perturbation. Prior to each measurement, the samples were kept at rest for 10 min in order to stabilize the temperature of the electrolytic medium and achieve quasi-steady-state conditions. Potentiodynamic polarization was performed from −1000 to +1000 mV at a scan rate of 10 mV/s, in order to obtain the full anodic and cathodic response of the bare substrate. In the second set, Mg-AZ91D samples coated with PHB were examined. For these, polarization was recorded over a more restricted potential window of −250 to +250 mV at the same scan rate. All tests were conducted in HANK’s solution (0.4 g/L of KCl, 8.0 g/L of NaCl, 1.0 g/L of glucose (C_6_H_12_O_6_), 0.06 g/L of KH_2_PO_4_, 0.048 g/L of Na_2_HPO_4_, 0.14 g/L of CaCl_2_, 0.098 g/L of MgSO_4_·7H_2_O, and 0.35 g/L of NaHCO_3_) maintained at 37 °C.

## 3. Results and Discussion

### 3.1. Surface Free Energy and Contact Angle of Bare and Treated Mg-AZ91D

**Figure 1:**
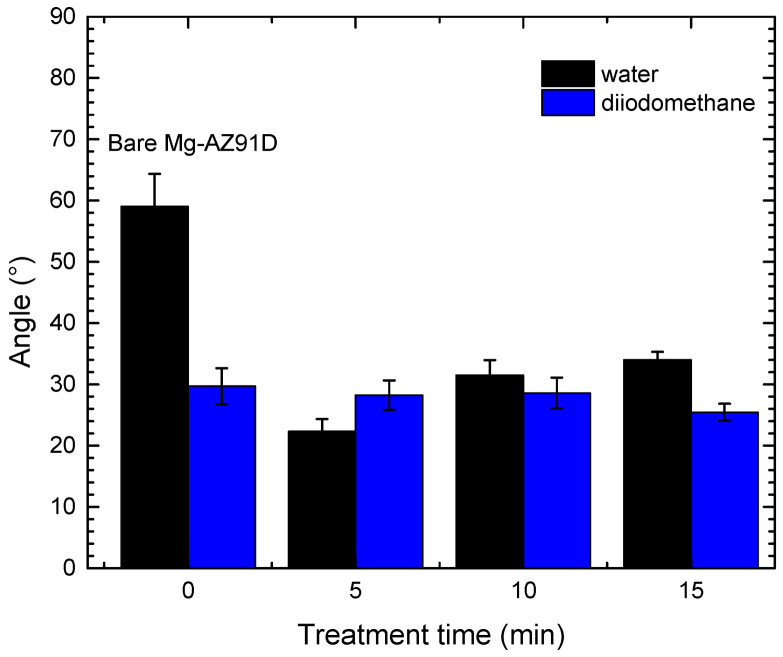
Contact angle of Mg-AZ91D.

As shown in Figure 1, bare Mg-AZ91D exhibited a contact water angle of 59°, indicating low surface wettability. After plasma treatment, all treated Mg samples showed a significant decrease in contact angle, with the most pronounced effect observed in the specimen treated for 5 min, which exhibited a value of 22°. This indicates a notable improvement in substrate wettability due to the plasma treatment. However, increasing the treatment time beyond 5 min resulted in a higher contact angle, suggesting a partial loss in this surface property.

**Figure 2:**
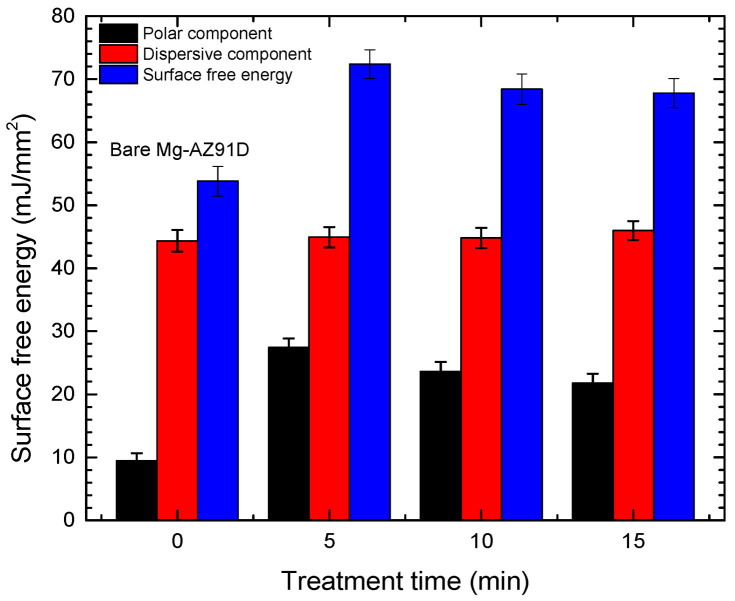
Surface free energy of Mg-AZ91D as a function of treatment time.

Figure 2 illustrates that the untreated Mg-AZ91D surface exhibited a surface free energy of 53.82 mJ/mm^2^. After plasma exposure, the polar component increased substantially, reaching its highest value in the sample treated for 5 min, which resulted in a total surface energy of 72.4 mJ/mm^2^. In contrast, the dispersive component remained practically unchanged. However, as observed for the contact angle measurements, treatment times longer than 5 min led to a reduction in surface energy. Therefore, a plasma treatment of 5 min can be considered optimal for promoting higher surface activity. Overall, these changes reflect modifications in both the wettability and surface chemistry of the metal.

### 3.2. Surface Free Energy and Contact Angles of PHB

**Figure 3:**
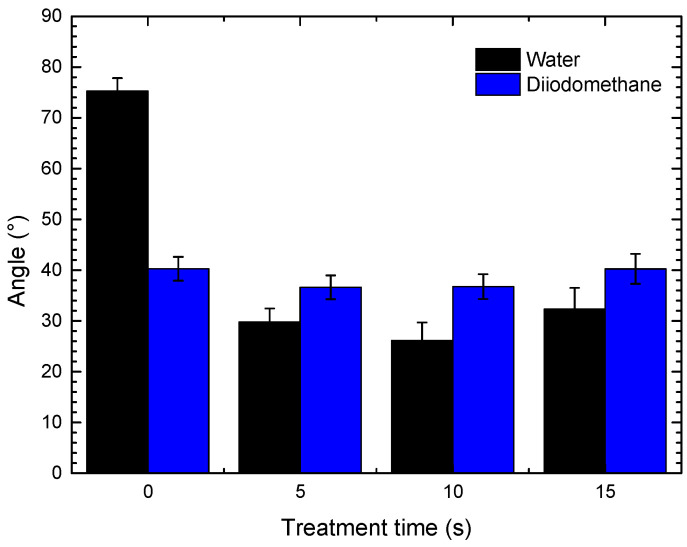
Contact angle of PHB.

Figure 3 shows the contact angles of the PHB coating as a function of plasma treatment time. The untreated coating exhibited a contact angle of 75° when measured with distilled water, consistent with reported values for PHB films, which have been characterized as materials with moderate hydrophobicity due to their high crystallinity and low surface polarity [11]. After plasma treatment, a significant reduction of up to 65% was observed in the contact angle, which decreased from 75° to 26° after only 10 s of treatment. This decrease indicates higher hydrophilicity relative to that of the untreated material. This effect is associated with the incorporation of polar functional groups (–OH, C=O, –COOH) on the surface of the coating, which increases affinity for water. Similar behavior has been reported in previous studies, where plasma treatment promoted the formation of oxygen-containing groups at the surface of materials, improving wettability. Enhancements in this property have been reported in plasma-treated PHB nanofibers, evidenced by a decreased contact angle and increased surface roughness [12].

**Figure 4:**
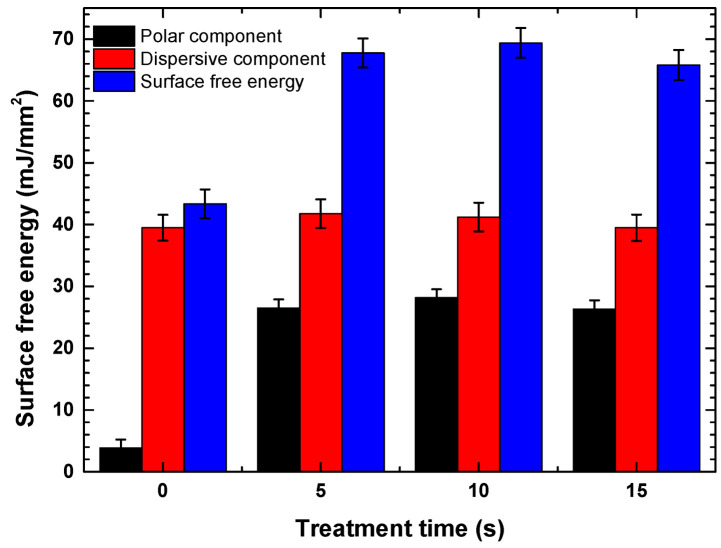
Surface free energy of PHB as a function of treatment time.

On the other hand, Figure 4 shows the surface free energy results as a function of treatment time. The untreated coating exhibited a surface free energy of 43.34 mJ/mm^2^. After plasma treatment for 5, 10, and 15 s, values of 67.75, 69.38, and 65.80 mJ/mm^2^ were recorded, respectively, evidencing significant surface activation. These results are consistent with the improved wettability observed through the reduction in contact angle and support the hypothesis that plasma treatment favorably modifies the surface chemistry of PHB.

### 3.3. Adhesion Test

**Figure 5:**
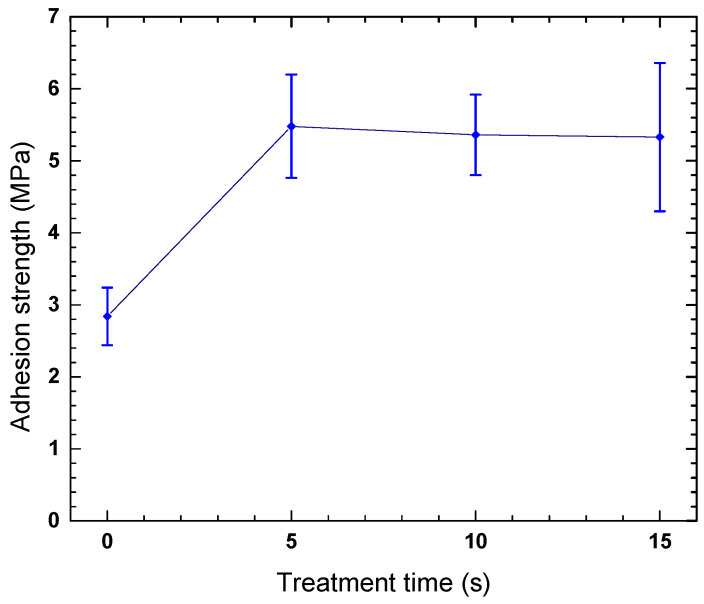
Adhesion of PHB coating on plasma-treated Mg-AZ91D substrate.

The results obtained from the pull-off test (Figure 5) demonstrate a significant improvement in the adhesion of the PHB coating on the Mg-AZ91D alloy after plasma treatment. Initially, a 5 min treatment was selected to functionalize the substrate surface, as this condition produced the greatest increase in surface free energy, as previously discussed. This elevated surface energy facilitated the formation of stronger bonds between the metallic substrate and the PHB coating.

The lowest adhesion strength value was observed for magnesium coated with untreated PHB, measuring 2.84 MPa. After treatment, an increase in the force required to detach the coating was observed, reaching a maximum value of 5.43 MPa after 5 s of plasma treatment. This increase is attributed to the introduction of polar functional groups on the PHB surface during plasma treatment, which enhanced the interaction between the polymer and the metallic substrate.

It is noteworthy that the substrate surface treatment was performed only under the 5 min condition, which was identified as the most effective for increasing the surface energy of magnesium based on previous contact angle and surface free energy results.

### 3.4. XRD Analysis of Mg-AZ91D Alloy and PHB

**Figure 6:**
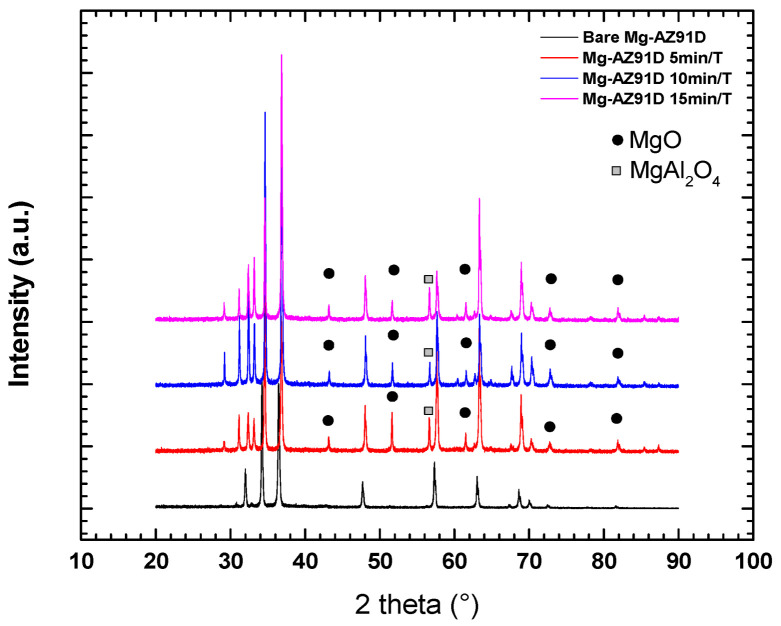
XRD pattern of Mg-AZ91D alloy with and without plasma treatment.

X-ray diffraction analysis revealed the main phases of the Mg-AZ91D alloy, including α-Mg (located at 2θ angles of 31°, 34°, 36°, 47°, 63°, and 70°) and β-Mg_17_Al_12_ (at 57° and 72°). After plasma treatment for 5, 10, and 15 min, peaks associated with MgO and MgAl_2_O_4_ appeared (Figure 6), due to reactive oxidizing species in atmospheric plasma, such as O_3_, O*, and O_2_^+^, which promote magnesium oxidation. This is consistent with a study of Mg-AZ91D produced by stir casting [13].

From the peak (101) of α-Mg at 2θ ≈ 36°, the full width at half maximum (FWMH, β) and the crystallite size were calculated using the Scherrer equation (Equation (1)), considering Cu Kα radiation (λ = 0.15406 nm) and a form factor K = 0.9 (Table 1).

The results show that the crystallite size increases significantly after 5 and 10 min of treatment with plasma at atmospheric pressure (from 39 nm in the sample without plasma to 71 and 81 nm, respectively), indicating an improvement in crystallinity. However, after 15 min of plasma treatment, the crystallite size decreases again to 39 nm, suggesting that longer exposure to plasma causes adverse effects, probably associated with the formation of oxides.

**Figure 7:**
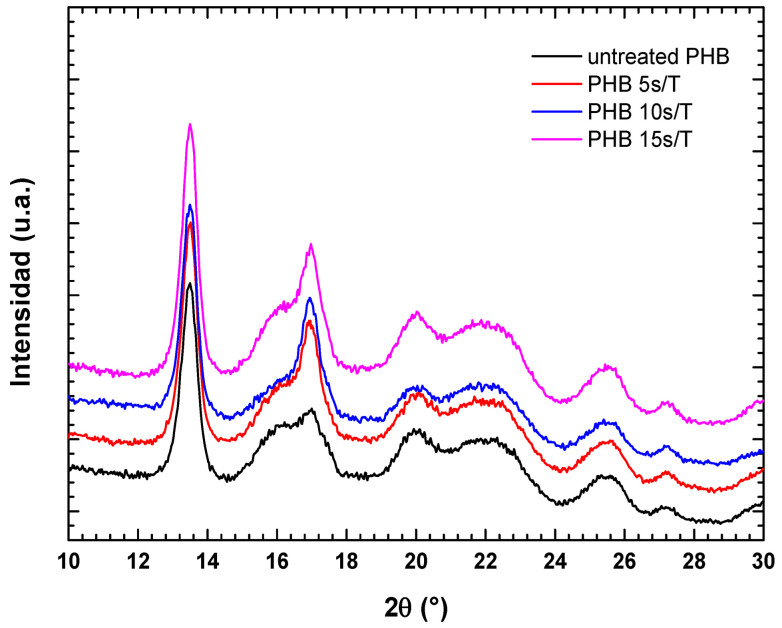
XRD patterns of untreated and plasma-treated PHB.

Figure 7 shows the diffraction patterns of PHB after no treatment and after plasma treatment for 5, 10, and 15 s. The diffractogram exhibits characteristic PHB peaks at approximate 2θ values of 13°, 17°, 20°, 22°, 25°, and 27°, corresponding to the crystallographic planes (020, 110, 101, 111, 121, and 040). This reflects the orthorhombic unit cell structure of PHB, inherent to its crystalline nature [14].

An increase in the FWHM and definition of the peak at around 2θ ≈ 13° is observed for untreated PHB; these increase with treatment time up to 10 s, which is related to an increase in polymer crystallinity. Additionally, the crystallite size (Xs) was calculated for the characteristic peaks (Table 2). The greatest size was observed for the PHB sample treated for 10 s. This increase in size, calculated using the Scherrer equation, is directly associated with the better organization of crystalline domains, indicating an increase in material crystallinity.

After 5 s of treatment, some planes improved, while others decreased; after 15 s, some values dropped compared to those after the 10 s treatment. This may have been due to the treatment time being too short or surface damage/degradation of the polymer, respectively. These results reveal that a 10 s plasma treatment is optimal for modifying not only the polymer surface but also its internal structure, improving its crystallinity.

### 3.5. Raman Spectroscopy of PHB

**Figure 8:**
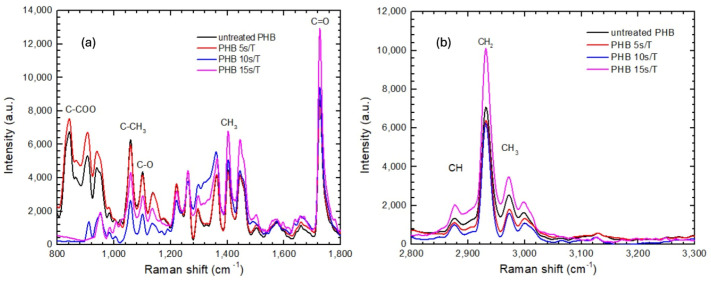
(**a**) Raman spectra of untreated and plasma-treated PHB, region from 800 to 1800 cm^−1^; (**b**) Raman spectra of untreated and plasma-treated PHB, region from 2800 to 3300 cm^−1^.

The Raman spectra of PHB that was untreated and plasma-treated for 5, 10, or 15 s exhibit characteristic peaks associated with the functional groups and molecular bonds of this polyester. The most relevant bands are those at 1725 cm^−1^, corresponding to the carbonyl (C=O) stretching of the ester groups, characteristic of polyhydroxyalkanoates; 1450 cm^−1^, associated with the deformation of methyl (CH_3_) and methylene (CH_2_) groups; 1100 cm^−1^, related to C–O and C–C bond vibrations; 1056 cm^−1^, attributed to C–O–C stretching (Figure 8a); and 2930 cm^−1^, corresponding to the asymmetric stretching of CH_3_ and CH_2_ groups (Figure 8b). These results are consistent with those reported in other studies on the spectroscopic properties of PHB [14,15].

Both the untreated PHB and the sample treated for 5 s show similar spectra. However, for samples treated for 10 or 15 s, an increase is observed in the bands at 800 and 900 cm^−1^, corresponding to C–C and C–O stretching. This suggests the incorporation of –OH, C=O, C–O–C, and –COOH groups as a result of plasma treatment, indicating surface functionalization of the polymer.

### 3.6. Potentiodynamic Polarization Curves and Electrochemical Impedance Spectroscopy of Mg-AZ91D

**Figure 9:**
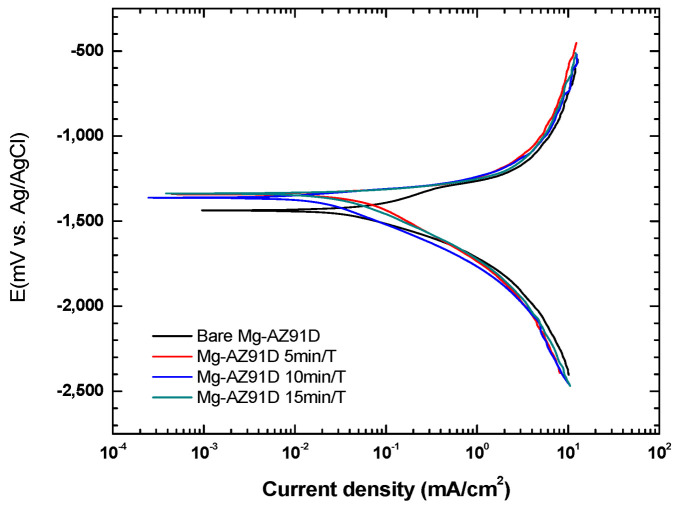
Potentiodynamic polarization curves of plasma-treated Mg-AZ91D.

Figure 9 shows the typical curves of Mg-AZ91D alloys in physiological solutions [16]. Our test evaluated both bare Mg-AZ91D and samples treated with atmospheric plasma to examine the protective effect of plasma. After treatment, the corrosion potential (Ecorr) gradually shifted toward more positive values, changing from −1438.9 mV in the untreated alloy to −1334.7 mV in the sample treated for 15 min (Table 3). This suggests a reduced tendency for corrosion to initiate. However, the corrosion rate (Vcorr) showed no significant change, staying around 4.08 mm/year for the bare alloy and about 3.64 mm/year for the plasma-treated samples, regardless of treatment time.

**Figure 10:**
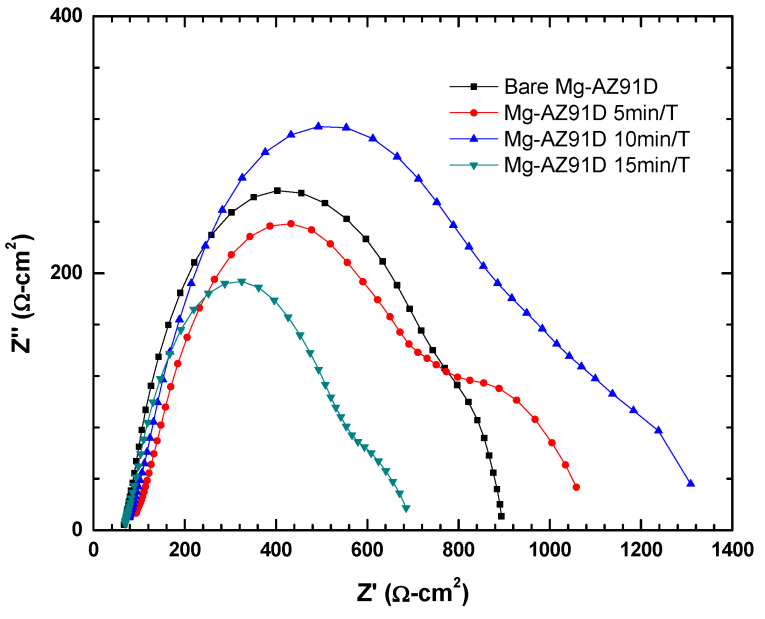
Nyquist plot of plasma-treated Mg-AZ91D.

**Figure 11:**
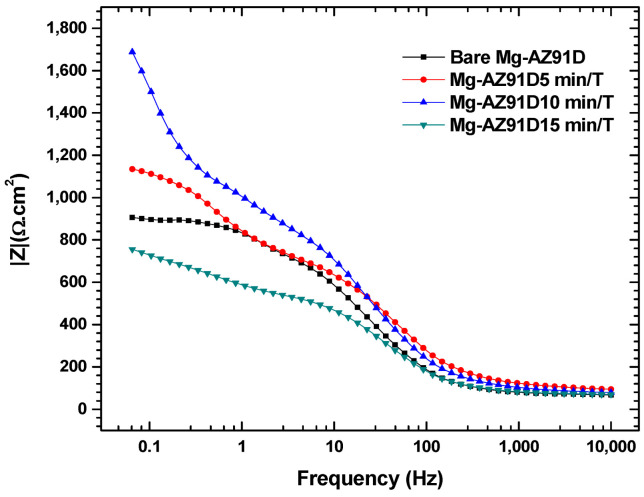
Impedance of plasma-treated Mg-AZ91D.

Figure 10 shows the electrochemical impedance spectroscopy results of Mg-AZ91D after different plasma treatment times. A semicircle is observed in all four conditions, which is characteristic of a charge transfer process [17]. Figure 11 reveals that the sample treated for 10 min exhibited the highest impedance, indicating the formation of corrosion products due to the implantation of radicals such as –OH, O, N, NO, and O_3_ from the atmospheric plasma [18]. These led to the formation of products like MgO, which can act as a slightly protective barrier against corrosive attack from solution ions. However, at 15 min, this charge transfer resistance decreased significantly, demonstrating that 15 min of plasma treatment may cause some wear, erosion, or formation of less protective corrosion product layers.

**Figure 12:**
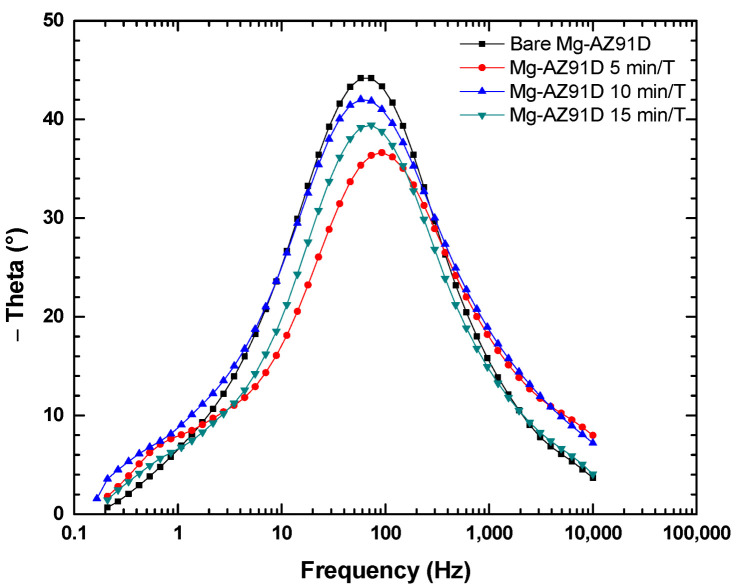
Bode phase plot of plasma-treated Mg-AZ91D.

Figure 12 shows a single time constant with a maximum phase angle at intermediate frequencies (10–1000 Hz), confirming that the dominant process is charge transfer at the Mg-AZ91D/solution interface [19].

### 3.7. Potentiodynamic Polarization Curves and Electrochemical Impedance Spectroscopy of PHB-Coated Mg-AZ91D

**Figure 13:**
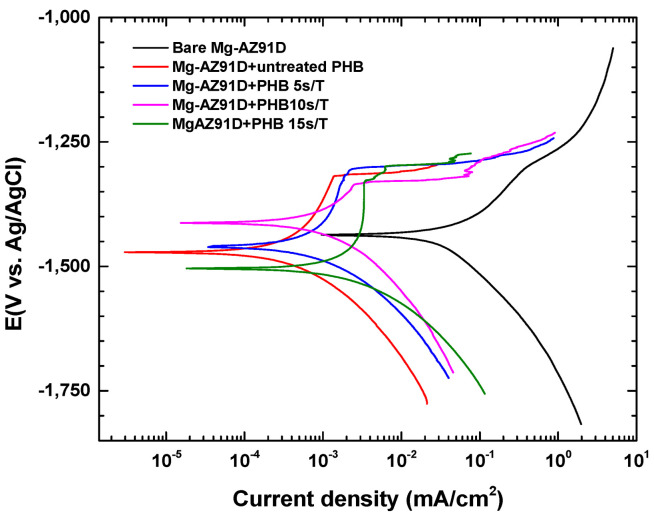
Potentiodynamic polarization curves of Mg-AZ91D and PHB-coated Mg-AZ91D.

Figure 13 shows the potentiodynamic polarization curves of bare Mg-AZ91D and Mg-AZ91D treated with plasma for 5 min and coated with PHB, which was also plasma-treated for 5, 10, or 15 s in HANK solution. The corrosion potential (E_corr_) shifts to more negative values, decreasing from −1438 mV for the bare magnesium to −1504 mV for Mg-AZ91D + PHB after 15 s of treatment. This suggests a stronger interaction between the working electrode and the electrolyte solution, confirming functionalization of the PHB coating due to atmospheric plasma, incorporating groups such as –OH, C=O, –COOH, –NH_2_, and –CONH_2_ [20].

As expected, the corrosion rate (V_corr_) shows a significant decrease of approximately 94% to 99% under all Mg-AZ91D + PHB conditions compared to that for the bare Mg-AZ91D (4.083 mm/year), corresponding to values ranging from 0.001 mm/year to 0.009 mm/year, as shown in Table 3. The protective performance of the coating was evaluated by calculating the protection efficiency (*ε_PDP_*) according to the equation below:

(2) $$\varepsilon_{PDP}\left(\%\right)=\frac{Icorr,susbtrate-Icorr,coating}{Icorr,substrate}\times 100$$

The porosity of the coating plays an important role in determining the protective performance of the PHB layer. In this study, porosity is quantified using the following expression:

(3) $$P=\frac{Rp(u)}{Rp(c)}\times 10^{-\frac{\left|∆Ecorr\right|}{Ba}}$$

where P represents the porosity, Rp(u) is the polarization resistance of the uncoated magnesium, and Rp(c) is that of the coated sample. ΔEcorr is the difference between the corrosion potentials of the uncoated and coated magnesium, and Ba is the anodic Tafel slope.

The polarization resistances Rp(u) and Rp(c) are calculated using the Stern–Geary equation:

(4) $$Rp=\frac{BaBc}{2.303\;Icorr(Ba+Bc)}$$

According to the results shown in Table 4, all coatings exhibited high levels of protection, exceeding 90%, with Mg-AZ91D + PHB 5 s/T standing out by displaying the highest polarization resistance and 99% coating efficiency, as well as the lowest corrosion rate (0.001 mm/year). Porosity is related to better coating efficiency since it minimizes pathways for the corrosive medium to reach the substrate, thereby slowing the corrosion process. This was confirmed in the Mg-AZ91D + PHB 5 s/T and Mg-AZ91D + PHB 15 s/T samples, which showed efficiencies of 98% and 99%, respectively, with porosities of 0.46% and 0.20%.

**Figure 14:**
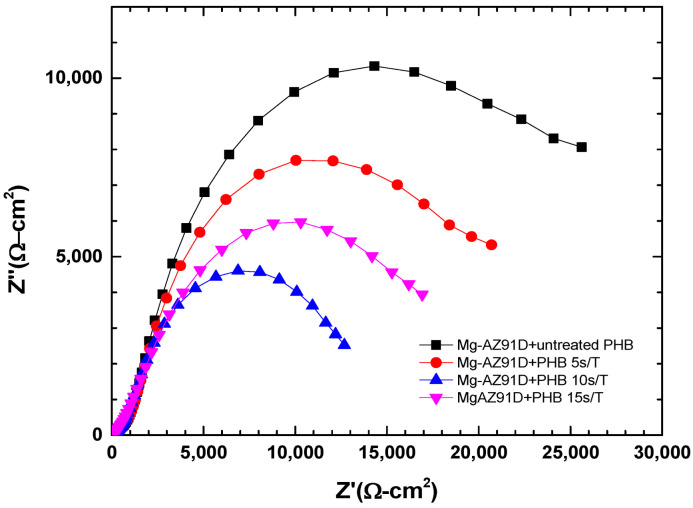
Nyquist plot of Mg-AZ91D coated with PHB.

**Figure 15:**
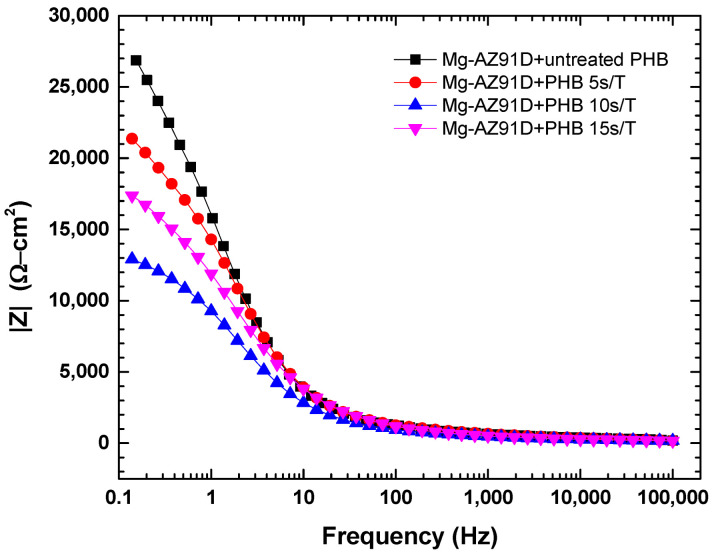
Impedance of Mg-AZ91D coated with PHB.

**Figure 16:**
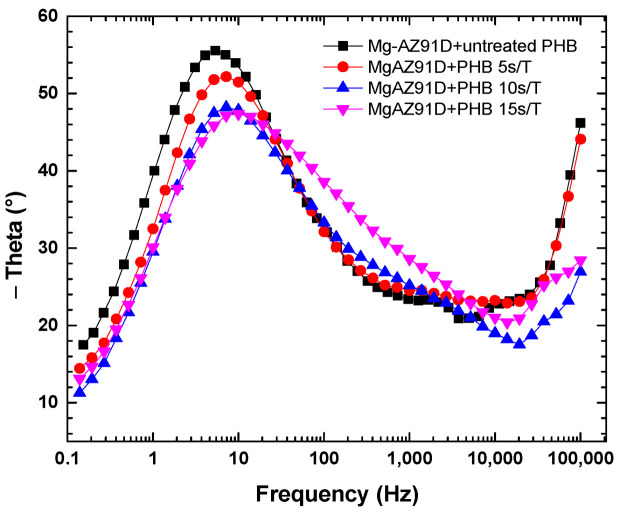
Bode plot of Mg-AZ91D coated with PHB.

The electrochemical impedance spectroscopy data for Mg-AZ91D are shown in Figure 14, Figure 15 and Figure 16. The Mg-AZ91D samples were first treated with atmospheric plasma for 5 min and then coated with PHB, which was subjected to plasma treatment for varying durations (5, 10, or 15 s). The Nyquist plots (Figure 14) show a single semicircle for each condition, which is mainly associated with the charge transfer process. However, the phase angle diagram (Figure 16) reveals two time constants, indicating the presence of two electrochemical processes with different characteristic times. The time constant at medium frequencies (1–1000 Hz) is attributed to charge transfer at the electrode/electrolyte interface, while that at high frequencies (10,000–100,000 Hz) corresponds to the barrier properties of the PHB coating, which acts as a protective film against corrosion [21,22,23].

## 4. Discussion

The present study demonstrates that atmospheric plasma treatment plays a critical role in tailoring the surface properties, interfacial adhesion, structural characteristics, and corrosion performance of the Mg-AZ91D/PHB coating system. The correlation between wettability, surface free energy, structural modifications, adhesion strength, and electrochemical behavior highlights the synergistic effect of plasma treatment on both the metallic substrate and the polymer coating.

Plasma treatment of Mg-AZ91D significantly enhanced surface wettability, as evidenced by the marked reduction in water contact angle and the corresponding increase in surface free energy. This enhancement was mainly driven by an increase in the polar component, indicating the introduction of oxygen-containing functional groups and surface oxides. XRD analysis corroborated these findings through the appearance of MgO and MgAl_2_O_4_ phases after plasma exposure, which are known to increase surface reactivity and promote chemical bonding. The increase in crystallite size observed after 5 and 10 min of treatment further suggests a plasma-induced reorganization of the surface structure. However, excessive treatment (15 min) resulted in reduced crystallite size and surface energy, indicating that prolonged plasma exposure can lead to surface degradation or excessive oxide formation, which negatively affects surface activity (see Table 5).

Similarly, plasma treatment of PHB resulted in a substantial decrease in contact angle and a pronounced increase in surface free energy, confirming effective surface activation. Raman spectroscopy revealed incorporation onto the PHB surface of polar functional groups such as –OH, C=O, and –COOH, while XRD analysis showed an increase in crystallinity, particularly for the sample treated for 10 s. These structural and chemical modifications enhance polymer–substrate interactions by increasing polarity and improving molecular organization at the interface. However, longer exposure times led to partial loss of these benefits, likely due to surface damage or polymer chain scission.

The combined plasma treatment of Mg-AZ91D (5 min) and PHB (5–15 s) resulted in a significant improvement in coating adhesion, with the highest pull-off strength obtained for PHB treated for 5 s. This behavior is strongly correlated with the optimal balance between surface activation and structural integrity. Increased surface energy of the substrate, together with functionalized polymer chains, promotes mechanical interlocking and chemical bonding at the interface. Excessive plasma exposure, although still beneficial compared to untreated samples, slightly reduced adhesion strength due to over-oxidation or microstructural changes. (see Table 6).

Electrochemical studies further confirmed the strong correlation between surface modification, coating integrity, and corrosion protection. While plasma treatment alone slightly improved the corrosion resistance of Mg-AZ91D by shifting the corrosion potential to more noble values and increasing impedance, it was insufficient to significantly reduce the corrosion rate. In contrast, the PHB-coated samples exhibited a dramatic reduction in corrosion rate (94–99%), highlighting the dominant protective role of the polymer coating. Among the coated systems, Mg-AZ91D + PHB treated for 5 s showed the highest polarization resistance, lowest porosity, and maximum protection efficiency (≈99%). EIS results revealed two time constants, corresponding to the coating barrier effect and charge transfer processes, confirming that plasma-treated PHB acts as an effective corrosion barrier while maintaining strong adhesion to the substrate.

Overall, the correlation of surface, structural, mechanical, and electrochemical analyses clearly indicates that optimal plasma treatment conditions are essential to maximize coating performance. Moderate plasma exposure enhances surface chemistry and interfacial bonding, whereas excessive treatment can compromise structural stability and functional performance (see Table 7).

Optimal adhesion is associated with adequate crystallinity of the polymer, since a balance between crystalline and amorphous regions provides mechanical rigidity and, at the same time, interfacial mobility. Moderate crystallinity increases the cohesion of the coating, while the amorphous regions favor humidification and interaction with the substrate. Plasma treatment contributes to improving adhesion not only by inducing changes in crystallinity, but also by modifying the surface of the material, generating effects that strengthen the interfacial union.

## 5. Conclusions

The conclusions of the research work can be summarized as follows:

1. Atmospheric plasma treatment effectively modifies the surface properties of Mg-AZ91D and PHB by increasing surface free energy and introducing polar functional groups, leading to enhanced wettability and surface reactivity.
2. An optimal plasma treatment time of 5 min for Mg-AZ91D and 5–10 s for PHB was identified, providing the best balance between surface activation and structural stability within the studied experimental.
3. XRD and Raman analyses confirmed that plasma treatment induces controlled structural and chemical modifications, including oxide formation on Mg-AZ91D and increased crystallinity and functionalization of PHB.
4. The improved surface chemistry directly translated into enhanced interfacial adhesion, with the highest pull-off strength observed for Mg-AZ91D treated for 5 min and PHB treated for 5 s.
5. Electrochemical tests demonstrated that while plasma treatment alone offers limited corrosion protection, the combination of plasma treatment and PHB coating provides outstanding corrosion resistance, reducing the corrosion rate by up to 99%.
6. The Mg-AZ91D + PHB (5 s plasma-treated) system exhibited the highest polarization resistance, lowest porosity, and maximum coating efficiency, confirming its superior protective performance.
7. The strong correlation between surface free energy, adhesion strength, microstructural changes, and electrochemical behavior highlights the effectiveness of atmospheric plasma treatment as a sustainable and efficient strategy for enhancing biodegradable polymer coatings on magnesium alloys.

In future work, the morphological characterization of the coatings will be deepened in order to correlate the surface changes with the adhesion properties. Likewise, the effect of different plasma treatment times will be evaluated to identify optimal surface modification conditions. In a complementary way, atomic force microscopy (AFM) analysis will be incorporated to study topography and roughness on a nanometric scale, which will allow a more comprehensive understanding of the adhesion mechanisms.

## Figures and Tables

**Table 1:** Grain size (D) calculated from the (101) peak of α-Mg according to Scherrer’s equation.

Plasma Time (min)	Peak (hkl)	2θ (°)	FWHM (°)	FWHM (rad)	Crystallite Size Xs (nm)
No plasma	(101)	36	0.21433	0.00374	39
Plasma 5 min	(101)	36.77	0.11806	0.00206	71
Plasma 10 min	(101)	36.23	0.10366	0.00180	81
Plasma 15 min	(101)	36.8	0.21383	0.00373	39

**Table 2:** Crystallite sizes of PHB.

2θ (°)	Plane	FWHM (rad)	Xs Untreated PHB (Ǻ)	FWHM (rad)	Xs PHB 5 s/T (Ǻ)	FWHM (rad)	Xs PHB 10 s/T (Ǻ)	FWHM (rad)	Xs PHB 15 s/T (Ǻ)
13	020	0.0084	164	0.0081	172	0.0081	172	0.0082	169
17	110	0.0092	151	0.0097	143	0.010	139	0.0095	146
20	101	0.0127	110	0.0118	118	0.0119	117	0.0118	118
22	111	0.0146	96	0.0187	75	0.0117	120	0.0158	89
25	121	0.0144	98	0.0141	100	0.0121	116	0.0133	106
27	040	0.0081	174	0.0073	192	0.0064	219	0.0073	192

**Table 3:** Electrochemical parameters of Mg-AZ91D treated with atmospheric plasma.

Condition	I_corr_ (mA/cm^2^)	E_corr_ (mV)	V_corr_ (mm/year)
Bare Mg-AZ91D	0.1787	−1438.90	4.083
Mg-AZ91D 5 min/T	0.1522	−1341.83	3.478
Mg-AZ91D 10 min/T	0.1522	−1359.61	3.645
Mg-AZ91D 15 min/T	0.1591	−1334.73	3.635

**Table 4:** Electrochemical parameters of Mg-AZ91D treated with plasma for 5 min + PHB as a function of treatment time: 5, 10, and 15 s.

Condition	I_corr_ (mA/cm^2^)	E_corr_ (V)	V_corr_ (mm/year)	Bc (V)	Ba (V)	Rp (kΩ-cm^2^)	P (%)	ε_PDP_ (%)
Bare Mg-AZ91D	1.7 × 10^−4^	−1.44	4.083	0.090	0.067	0.09	N/A	N/A
Mg-AZ91D + untreated PHB	9.4 × 10^−6^	−1.47	0.009	0.085	0.066	1.72	14.87	94
Mg-AZ91D + PHB 5 s/T	1.7 × 10^−6^	−1.46	0.001	0.085	0.066	9.50	1.80	99
Mg-AZ91D + PHB 10 s/T	6.0 × 10^−6^	−1.41	0.006	0.085	0.066	2.69	1.19	96
Mg-AZ91D + PHB 15 s/T	3.4 × 10^−6^	−1.50	0.003	0.085	0.066	4.75	14.89	98

**Table 5:** Correlation between plasma treatment, surface properties, and adhesion behavior of Mg-AZ91D/PHB.

Plasma Treatment Condition	Surface Free Energy Trend	Wettability (Contact Angle)	Surface Chemistry/Structure	Adhesion Strength	Correlated Effect
Bare Mg-AZ91D + untreated PHB	Low	Poor wettability	Limited polar groups, native oxide	Lowest (2.84 MPa)	Weak interfacial bonding due to low surface activity
Mg-AZ91D (5 min) + untreated PHB	High (polar component)	Improved	Formation of MgO/MgAl_2_O_4_, increased crystallite size	Moderate	Enhanced substrate reactivity improves adhesion
Mg-AZ91D (5 min) + PHB 5 s/T	Very high	Excellent	Polar functional groups on PHB; optimal surface activation	Highest (5.43 MPa)	Strong chemical bonding and mechanical interlocking
Mg-AZ91D (5 min) + PHB 10 s/T	High	Very good	Increased PHB crystallinity and functionalization	Slightly reduced	Over-functionalization slightly reduces adhesion
Mg-AZ91D (5 min) + PHB 15 s/T	Moderate	Reduced compared to optimum	Possible surface degradation	Lower than optimum	Excess plasma exposure weakens interfacial strength

**Table 6:** Correlation between plasma-induced structural modifications and surface chemistry.

Material	Plasma Time	Structural Change (XRD)	Spectroscopic Evidence	Surface Effect	Overall Implication
Mg-AZ91D	5 min	Increased crystallite size	MgO, MgAl_2_O_4_ formation	Increased polarity	Optimal surface activation
Mg-AZ91D	10 min	Further crystallite growth	Enhanced oxide formation	Stable surface	Improved corrosion impedance
Mg-AZ91D	15 min	Crystallite size reduction	Excessive oxidation	Surface degradation	Reduced protective efficiency
PHB	5 s	Slight crystallinity improvement	Minimal chemical change	Good wettability	Effective surface activation
PHB	10 s	Highest crystallinity	Increased –OH, C=O, –COOH	Maximum surface energy	Optimal functionalization
PHB	15 s	Reduced crystallinity	Possible chain scission	Partial degradation	Diminished performance

**Table 7:** Global correlation between plasma treatment parameters and functional performance.

Plasma Treatment Strategy	Surface Activation	Adhesion	Structural Stability	Corrosion Protection	Overall Performance
No plasma	Low	Poor	Stable	Very poor	Unsuitable
Substrate plasma only	Moderate	Moderate	Stable	Limited	Partial improvement
Polymer plasma only	Moderate	Moderate	Stable	Good	Incomplete protection
Substrate (5 min) + PHB (5 s)	High	Excellent	Stable	Outstanding	Optimal system
Excess plasma exposure	Reduced	Decreased	Degraded	Reduced	

## Data Availability

Data is contained within the article.
